# Evaluation of Enkephalin-Degrading Enzymes in Sperm from Heroin-Addicted Men

**DOI:** 10.22074/ijfs.2020.5817

**Published:** 2019-11-11

**Authors:** Samira Rezaei-Mojaz, Zohreh Nazmara, Mohammad Najafi, Mansoureh Movahedin, Zahra Zandieh, Peymaneh Shirinbayan, Mohsen Roshanpajouh, Hamid Reza Asgari, Mehdi Abbasi, Morteza Koruji

**Affiliations:** 1 Department of Anatomical Sciences, School of Medicine, Iran University of Medical Sciences, Tehran, Iran; 2 Cellular and Molecular Research Center, Iran University of Medical Sciences, Tehran, Iran; 3 Department of Biochemistry, School of Medicine, Iran University of Medical Sciences, Tehran, Iran; 4 Anatomical Sciences Department, School of Medicine, Tarbiat Modares University, Tehran, Iran; 5 Shahid Akbarabadi Clinical Research Development Unit (ShACRDU), Iran University of Medical Sciences, Tehran, Iran; 6 Pediatric Neuro-Rehabilitation Research Center, The University of Social Welfare and Rehabilitation Sciences, Tehran, Iran; 7 School of Behavioral Sciences and mental Health, Tehran Psychiatry Institute, Iran University of Medical Sciences, Tehran, Iran; 8 Department of Anatomy, School of Medicine, Tehran University of Medical Sciences, Tehran, Iran

**Keywords:** Addiction, Aminopeptidase N, Endopeptidase, Heroin, Sperm Quality

## Abstract

**Background:**

The aim of this study was to investigate two enkephalin-degrading enzymes, aminopeptidase N (APN/ CD13) and endopeptidase (NEP/CD10), gene and protein expression levels in sperm samples of fertile and heroin- addicted men, and the correlation between their expressions and semen quality.

**Materials and Methods:**

In this case-controlled study, semen was collected from 24 normozoospermic healthy (as a control group) and 24 heroin-addicted men donors (as case or addiction group). Sperm cells isolated by Cook Medical gradient (40-80%) and followed up by swim-up techniques were used for real-time quantitative polymer- ase chain reaction (qPCR) and flow cytometry techniques to assess APN/CD13 and NEP/CD10 genes and proteins subsequently. Semen parameters were analyzed by computer-assisted sperm analysis.

**Results:**

The findings revealed that there were significant differences in sperm total motility (41.07 ± 3.63 vs. 63.03 ± 3.31 %, P=0.0001), progressive motility (35.21 ± 2.64 vs. 20.93 ± 3.22%, P=0.001) and viability (69.9 ± 4.69 vs. 86.81 ± 1.26 %, P=0.002) in the addicted group vs. control ones. APN and NEP gene expression levels in the addicted group decreased compared with the control ones (1.00 ± 0.67 vs. 0.36 ± 0.13, P= 0.008 and 1.07 ± 0.11 vs. 0.52 ± 0.12 0.002, respectively). Flow cytometry analysis showed that the average percent of APN/CD13 in heroin consumers significantly decreased compared with the healthy ones, while NEP/CD10 rate between two groups was similar. We also observed that duration of drug dependence is correlated with sperm viability (r=-0.627, P=0.016) and motility (r=-0.410, P=0.05), NEP (r=-0.434, P= 0.049), and APN (r=-0.641, P=0.002) gene expression levels.

**Conclusion:**

We conclude that semen quality and enkephalin-degrading enzymes were altered in heroin-addicted men. other confirming the internal validity of our estimates.

## Introduction

Infertility is one of the most serious social problems that has an effect on a huge percentage of couples. In general, the basic cause of infertility refers to the male partner in approximately 40-45% of cases (1). In some national data and available sources, the number of drug abusers in Iran is estimated to be between 1,200,000 and 2,000,000 people (2-4) with a mean age of 33 years (5). This means that they are generally young in reproductive age. Heroin, from the opioid group, is morphine O-acetylated at position 3 and 6 (diacetylmorphine) (6). Heroin addiction has been linked to some degenerative diseases including aging, abscesses, arthritis, other rheumatologic disorders, and immunological disorders (7).

The activity of the opioid system is mediated by endogenous opioid peptides (EOPs). EOPs or opiate alkaloids carry out their function through three types of opioid receptors [the delta-opioid receptor (DOR), the mu opioid receptor (MOR) and the kappa opioid receptor (KOR)] on membranes (8, 9). EOPs control male reproductive function at different levels (1): i. At the hypothalamus, they inhibit the secretion of gonadotropinreleasing hormone (GnRH) and suppress the rate of luteinizing hormone (LH) from the pituitary gland (decrease the levels oftestosterone), ii. At the testes, leydig cells produce EOPs through LH affect, and these peptides perform an inhibitory effect on sertoli cells [inhibiting the production of Androgen-binding protein (ABP)] and iii. In germ cells, somatic cells of the testes and iv. In sperm cells (regulates sperm motility). As a result, EOPs may be involved in human reproductive function by a direct effect on sperm. However, EOP levels are controlled by enzymatic degradation by aminopeptidase N (APN) and endopeptidase neutral N (NEP). These enzymes are present in both sperm and seminal fractions (10), and their activity in semen is particularly high compared with other body tissues (11). Interestingly, APN activity levels were found to be altered in semen from sub-fertile patients, suggesting that this enzyme may play an important role in male fertility (12). 

The effect of opiates on spermatozoa in some aspects is still unknown or controversial. Some studies have been performed on the effect of certain drug abuse on the human and mouse sperm parameters (13-15). In our previous study, we showed the deleterious effects of kerack, (unlike to crack cocaine), consumption in Iran on testis structure, sperm parameters, and particularly sperm morphology in the adult mouse. Also, It down-regulated the expression of CatSper genes, resulting in depression of sperm motility (16). Nazmara et al. (17) reported that heroin is strongly associated with abnormalities in histone-to-protamine transition and with human semen quality, particularly sperm morphology and motility.

Sperm motility is considered a key functional parameter that controls reproduction (18) and is widely used as an indicator of semen quality (19) since sperm must move to reach the oocyte and then penetrate it using sperm movement. More reports on heroin (13) and nicotine (20) consumption in animals and opioids in human (14, 17) showed decreasing in sperm parameters. 

Since decreased motility (asthenozoospermia) is a common abnormality among opiate drug addicts (17), it is likely that the opioid system involves the sperm movement. Previous studies have suggested expression of enkephalin-degrading enzymesin human spermand semen (1, 10, 12, 21). Subiran et al. (10) reported Enkephalindegrading enzymes were expressed in human sperm including messenger RNA of both enzymes and APN and NEP, (in a small number of sperms), were detected in spermatozoa at the protein level. In addition, the activity of the enkephalin-metabolizing enzyme aminopeptidase N was measured in various fractions of human semen from normal and subfertile patients by Irazusta et al. (12). They reported that the activity of aminopeptidase N was lower in males with asthenozoospermia as compared with normal semen. It has been described that the inhibition of some of those enzymes can significantly improve sperm motility (21). In the present study, we investigated the expression of two enkephalin-degrading enzymes APN and NEP in heroin-users’ sperm cells to assess any correlation between expression of abovementioned and sperm motility.

## Materials and Methods

### Participants

In this case-control study, participants were interviewed after written informed consent. The data on personal information (e.g. name, age, marital and parental status), history of addiction [duration of heroin consumption, heroin use (mg/day), cigarette smoking, and alcohol drinking], and medical status (e.g. medications, special illness and surgery) were obtained via a structured questionnaire.

Based on the medical files and questionnaires, twentyfour 20-50-year-old men with normal body mass index (BMI) who just used heroin for at least 12 months -without using other drugs during that interval- were selected as a case or heroin-addicted group. They were introduced from addiction treatment centers before entering treatment programs and should have met the Diagnostic and Statistical Manual of Mental Disorders (DSM-V) criteria for addiction.

Also, 24 age-BMI-matched men with a normal semen analysis according to World Health Organization (WHO) 2010 criteria volunteered to participate in this study as a control group. They were male partners of married couples without any illicit drug use who had attended the Shahid Akbar-Abadi Obstetrics and Gynecology Hospital of the Iran University of Medical Science for female infertility consultation

Subjects with medical problems known to be associated with subfertility in both groups, illicit-drug usersin control group, and individuals that started treatment with other drugs in addicted ones were excluded from the study.

### Sperm preparation

Semen samples were obtained from donors by masturbation after 2 to 3 days of abstinence into sterile containers and allowed to liquefy at 37˚C for 30 minutes before processing. Semen volume, as well as sperm concentration, viability (by eosin B staining), morphology (by Papanicolaou staining method), and motility were measured in each sample by CASA (22).

For eosin B staining (0.5% in saline), 20 microliters the sperm suspension was mixed with 7 µl eosin and observed under a light microscope (×400 magnification). Then, 200 sperm were counted and the percentage of live spermatozoa was recorded. With the staining, red sperm heads were considered as dead (23, 24).

Sperm morphology was assayed by the Papanicolaou method. First, smears were prepared and stained with the Papanicolaou method based on the protocol. Then, the morphology of 200 spermatozoa was surveyed under ×1000 oil immersion lens. With the staining, the nuclei turn blue, and the acrosome and tail become pink. Abnormal morphology wasreported in five fields of vision randomly, and the percentage of abnormal morphology was recorded (16). 

Before RNA extraction or flow cytometry, spermatozoa were isolated from semen on cook medical gradient (40-80%), followed by a swim-up in washing medium supplemented by Albumin 10% to recover motile cells without any contaminant leukocytes or other debris, and then they were visually examined under a light microscope. 

### Real-time quantitative polymerase chain reaction technique

All primers (Table 1) were designed by primer 3 (Online: Http://primer3.sourceforge.net), and then, primers were blasted in NCBI. 

**Table 1:** Sequences of the designed primers used for real-time quantitative polymerase chain reaction


Gene	Primer sequence (5ˊ-3ˊ)

*APN*	F: CCACCTTTCTGACATTGCC
	R: CAGGGGCCTGTACGTTTTTA
*NEP*	F: GCCTCAGCCGAACCTACAAG
	R: AGTTTGCACAACGTCTCCAAG
*β-actin*	F: GCAAGCAGGAGTATGACGA
	R: CAAATAAAGCCATGCCAATC


The RNA of spermatozoa was isolated with the RNeasy Mini kit (Qiagen, Germany). First strand cDNA synthesis was carried out using QuantiNova Reverse Transcription Kit (Qiagen, Germany). Then real-time quantitative polymerase chain reaction (qPCR) was performed using QuantiNova SYBR Green PCR Kit (Qiagen, Germany). 

The thermal cycling program included an initial incubation at 95˚C for 2 minutes, followed by 60 cycles of 95˚C for 5 seconds and 60˚C for 30 seconds. Three replicates of each reaction were performed, and the cycle threshold (Ct) values were averaged. Expression values were normalized to the average expression of the housekeeping gene (*β-actin*) and compared with a calibrator (control group) by the comparative Ct method (2^-∆∆ct^) (10).

### Flow cytometry analysis

Flow cytometry was performed on a BD Biosciences FACSCalibur in order to rate of presences of surface expression of enkephalinase and aminopeptidase on sperm cells. Sperm was stained directly for APN and NEP. Briefly, 1×10^6^ sperm in 2 ml phosphate buffered saline (PBS, Sigma, USA) was centrifuged in 4˚C for 10 minutes (×300 g). The pellet was suspended in 100 µl washing medium and then was added 20 µl antibody [PE Mouse Anti-Human CD13 and PE Mouse Anti-Human CD10 (BD Biosciences USA)]. Samples were incubated at 4˚C for 1 hour. Next, sperm was centrifuged three times at 4˚C for 10 minutes (×300 g). Finally, the pellet was suspended in 300 µl PBS and analyzed using the flow cytometer.

### Statistics analysis

Statistical analysis was done with statistical software (Ver. 16.0, Chicago, SPSS Inc.). The normal distribution was evaluated with the Kolmogorov-Smirnov test. The results were analyzed by performing independentsamples t test and Mann-Whitney U test. P≤0.05 was considered as statistically significant and mean ± SE was also calculated for each variable. The partial correlation and multiple regression analyses were done between APN and NEP levels and other parameters. 

### Ethical considerations

This study has been approved by the medical Ethics Committee of Iran University of Medical Science (code: IR. IUMS.rec.1394.9211313202). All human trails were carried out in accordance with the Declaration of Helsinki guidelines.

## Results

### Demographic information

Twenty-four healthy and twenty-four men addicted to heroin participated in the study. The mean ages in addicted and control groups were 35.44 ± 1.3 years and 33.91 ± 1.79 respectively, which there was no significant difference in average age. Although the range of BMI was normal in both groups, this parameter in the addicted men (22.34 ± 0.38 kg/m^2^) was significantly lower than the healthy ones (28.69 ± 0.91 kg/m^2^) (P≤0.01). All subjects in the addicted group were smoker, so there was a statistical difference between groups in this case (P≤0 .01).

### Semen quality analysis

According to our data, although semen volume, sperm concentration, and normal morphology were the same between groups, there was a significant decrease in sperm viability and motility rates in the addicted group (Table 2). 

**Table 2:** Sperm parameters in the study population


Parameters	Healthy men	Heroin addicted men	P value

Semen volume (ml)	3.63 ± 0.42	3.22 ± 0.35	0.457
Sperm concentration (×10^6^/ml)	115.79 ± 16.5	113.51 ± 18.9	0.928
Sperm total motility (%)	63.03 ± 3.31	41.07 ±3.63	0.0001
Sperm progressive motility (%)	35.21 ± 2.64	20.93 ± 3.22	0.001
Sperm normal morphology (%)	9.48 ± 1.54	12.11 ± 1.52	0.233
Sperm viability (%)	86.81 ± 1.26	69.9 ± 4.69	0.002


Data are presented as mean ± SE.

### Changes in enkephalinase and aminopeptidase genes expression 

As shown in the Table 3, *APN* and *NEP* gene expression levels in the addicted group (0.36 ± 0.13, 0.52 ± 0.12) decreased compared with the control ones (1.00 ± 0.67, 1.07 ± 0.11) (P≤0.01)

**Table 3:** Enkephalinase and aminopeptidase gene expression levels in healthy and heroin addicted men


Gene expression level	Healthy men	Heroin addicted men	P value

*APN* (2^-∆∆Ct^)	1.00 ± 0.67	0.36 ± 0.13	0.008
*NEP* (2^-∆∆Ct^)	1.07 ± 0.11	0.52 ± 0.12	0.002


Data are presented as mean ± SE.

### Flow cytometry analysis

**Fig 1:**
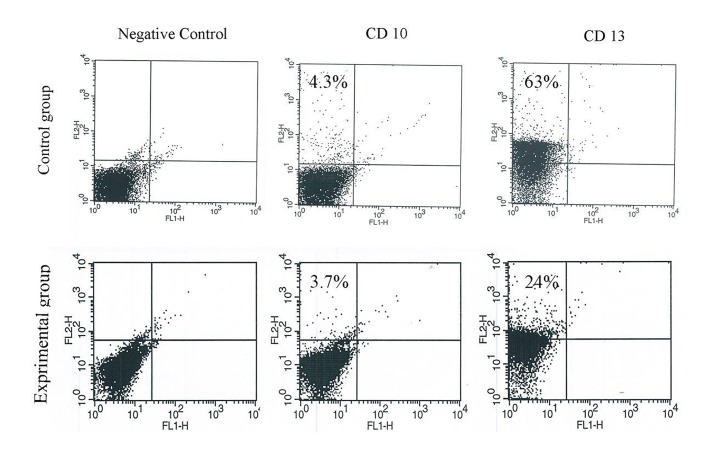
Flow cytometry analysis. It showed that the average percent aminopeptidase N (APN/CD13) in control group (63 ± 12%) significantly decreased compared to heroin addicted ones (24 ± 12%), while the average percent endopeptidase (NEP/CD10) in control (4.3 ± 1%) and addicted group (3.7 ± 2%) showed no significantly decrease.

Figure 1 shows flow cytometry analysis of cell surface protein enkephalinase and aminopeptidase in sperm of healthy and addicted men. The results demonstrated that the mean APN/CD13 rate in the control group (63 ± 12) was significantly higher than addicted ones (24 ± 12) (P≤0.05), while NEP/CD10 ratio in the control (4.3 ± 1) and addicted (3.7 ± 2) group did not show a significant difference.

### Correlation between enkephalinase and aminopeptidase genes expression and sperm motility 

As shown in Table 4 multivariate regression analysis proved that total motility is significantly correlated to duration of heroin dependence, whereas did not relate to other demographic data like age, BMI, cigarette smoking, and the amount of heroin daily use. Based on partial correlation test, sperm viability (r=-0.627, P=0.016) and total motility (r=-0.410, P=0.50), NEP (r=-0.434, P=0.049) and *APN* (r=-0.641, P=0.002) gene expression levels were significantly negative correlation with duration of heroin dependence (Table 5). 

**Table 4:** Correlation between motility and demographic data


Parameters	Unstandardized coefficients		Standardized coefficients	P value
		Beta	SE		Beta	

Age (Y)	1.221	0.731	0.437	0.108
BMI (kg/m^2^)	-0.262	1.082	-0.056	0.811
Duration of heroin consumption (Y)	-1.650	0.750	-0.799	0.040
Heroin use (mg/day)	1.821	2.468	0.134	0.468
Cigarette smoking	-0.427	10.109	-0.448	0.658


BMI; Body mass index and SE; Standard error.

**Table 5:** Partial correlation between duration of dependence and other studied parameters


Parameters	r-value	P value

Sperm viability (%)	-0.627	0.016
Sperm total motility (%)	-0.410	0.050
APN (2^-∆∆Ct^)	-0.641	0.002
NEP (2^-∆∆Ct^)	-0.434	0.049


Adjusted with cigarette smoking. APN; Aminopeptidase and NEP; Neutral endopeptidase.

## Discussion

The present study showed that enkephalin-degrading enzymes (NEP and APN) and sperm viability and motility were reduced in men addicted to heroin. We also showed that there was a significant negative correlation between *NEP* and *APN* gene expression levels with the duration of heroin dependence.

Infertility is one of the most important problems of human societies throughout the world. Considering the role of recreational heroin consumption as an idiopathic etiology of male infertility and increasing consumption of illicit drugs, especially among young people of reproductive age, socio-medical studies on this issue has been done less yet. The living conditions, lack of cooperation, simultaneous use of various drugs and legal and ethical problems in sampling in addicted people make the research difficult and complicated in this area. Therefore, research in this area can be very valuable. Our findings suggest a remarkable association among heroin addiction, asthenozoospermia and decreased APN and NEP mRNA levels. In addition, the duration of drug dependence is one of the main factors contributing to the detrimental effects of heroin on impaired male fertility.

Decreased in heroin users BMI may be caused by caloric malnutrition (25), inhibition of androgen production (26), or disorders of the gastrointestinal tract (27). Although our finding appears to be consistent with a number of studies (16, 26, 27), Diamond et al. (25) showed drug and alcohol abuse did not change the BMI in adolescent males. The average BMI may affect spermatogenesis. However, multivariate regression analysis showed that duration of heroin consumption can be more effective than BMI in semen parameters.

In this study, reduced sperm motility was observed in addicted men. Our finding is in line with other studies who mentioned that opioids such as heroin, kerack, and morphine can impair sperm parameters in mice and human. They also showed those alterations were dose-dependent (8, 13, 14, 16, 17). The opioid system likely influences reproductive function by the central nervous system (28), the pituitary gland, and the testis (29), exerting a direct action on the spermatozoa (30).

Reduced total and progressive sperm motility may be caused directly by heroin because of alteration of the encephalin-degrading enzymes. Researchers scrutinized the presence of APN/CD13 in the sperm head, neck and along the tail (19, 31). APN/CD13 was acknowledged to play a critical role in the sperm binding to oocyte due to being in the sperm head and control its motility by existence along the tail and in the neck. In the fact that opioid levels in semen are in charge of degrading enzymes like APN/ CD13, alteration of these enzymes could regulate sperm motility. Indeed, an adequate level of enkephalin as a delta opioid agonist is essential to sperm motility, but this effect depends on opiate concentrations (10). We proposed that heroin directly affects sperm motility by two mechanisms; first, a higher concentration of mu opioid agonists not only bind mu opioid receptors but also have an affinity to delta-opioid receptors. Heroin bind to the receptors occupies the enkephalin-binding site on the sperm and could deactivate the receptors and cause to accumulate opioids in semen. Second, Reducing the *APN* gene and subsequently, its protein in heroin consumers may result in opioid accumulation in the semen microenvironment. Indeed, the occupation of receptors and degrading enzymes deficiency or inactivity may affect the sperm quality and male fertility. Agirregoitia et al. (8) reported the inhibitory effect of the delta-antagonist naltrindole on sperm motility. 

Notwithstanding the level of *NEP* gene expression was statistically different between groups, its protein amount was similar in addicted and healthy men. In addition, a significant negative correlation between the *NEP* gene level and BMI was observed. Our data was proved by previous studies (10, 32, 33). Given that just a few cells after capacitation express NEP protein on sperm membranes (32), could justify the same protein amount between groups. This similarity may be due to the fact that NEP act by a mechanism that does not involve the opioid system which was suggested by Subiran et al. (10) who revealed naloxone does not affect sperm motility when incubated with thiorphan an encephalin degradation inhibitor for NEP. Our data showed that the amount of *NEP* gene and protein do not relate to each other. We hypothesize that this may be due to: i. Epigenetic regulators or the presence of NEP mRNAs in the profile of RNAs’ mature spermatozoa that interfere with embryogenesis; or ii. Since spermatozoa have a complex RNA package (34, 35), sperm cells could retain NEP messenger RNA for carrying epigenetic information in the zygote.

Based on the literature, NEP is the main peptidase that can hydrolyze tachykinins, which are present in spermatozoa and interfere in the regulation of sperm motility (12, 36, 37). Thus, alterations of NEP can cause slowly developing changes in sperm motility. Opioids and tachykinins, as bioactive peptides, could operate as signal molecules between spermatozoa and their environment, acting in an autocrine and/or paracrine fashion (38).

In addition, heroin can impair semen quality and alter sperm microenvironment by semen acidification and leukocytospermia (17), which probably affects the structure and function of these surface-expressed enzymes and influences semen parameters. 

The main limitation in the present study were: i. Sampling of heroin-addicted men was complicated because (a) heroin consumers usually take various addiction and narcotic drugs like opiate, kerack or methamphetamines, (b) the majority of addicted men had no trends to provide semen. Hence, we selected persons who used only heroin for at least 12 months -without using other drugs, ii. In addition; cigarette smoking was a major interference variable, which was controlled by partial correlation, iii. Scio-economic and family backgrounds were different among the participants. For example, diet may affect sperm parameters, and iv. We could not follow up the fertility status after treatment.

## Conclusion

We conclude that semen quality and enkephalindegrading enzymes were decreased in heroin-addicted men and there is a significant negative correlation between *NEP* and *APN* gene expression levels with the duration of heroin dependence. This study may increase our understanding of the effects of drugs and toxins on male infertility.
